# Diagnostic accuracy of dynamic contrast-enhanced CT for meningioma subclassification and its role in evaluating tumor vascularity

**DOI:** 10.1097/MD.0000000000048677

**Published:** 2026-05-15

**Authors:** Shanmei Liu, Tianchun Wang, Wei Li, Haitao Lu

**Affiliations:** a Department of Radiology, Changzhou No. 7 People’s Hospital, Changzhou City, Jiangsu Province, China; b Department of Radiology, The Third Affiliated Hospital of Soozhow University, Changzhou City, Jiangsu Province, China; c Department of Radiology, Beijing Hospital, Beijing, China; d Department of Radiology, The Third Affiliated Hospital of Soochow University, Changzhou City, Jiangsu Province, China; e The First People’s Hospital of Changzhou, Changzhou City, Jiangsu Province, China.

**Keywords:** blood volume, dynamic contrast-enhanced computed tomography, mean transit time, meningioma, perfusion imaging, permeability surface area product, subtype classification

## Abstract

Dynamic contrast-enhanced computed tomography (DCE-CT) has the potential to provide quantitative information on meningioma vascularity beyond conventional morphologic imaging. This retrospective study included 68 patients with histologically confirmed intracranial meningioma who underwent preoperative DCE-CT between October 2020 and October 2024. Quantitative perfusion parameters, including blood flow (BF), blood volume (BV), mean transit time (MTT), permeability surface area product (PS), time-to-peak enhancement, and arterial arrival time, were extracted from regions of interest drawn in the solid tumor portion and contralateral normal-appearing brain. Perfusion characteristics among histopathological subtypes and the diagnostic performance of these parameters for differentiating atypical (World Health Organization Grade II) from typical (World Health Organization Grade I) meningiomas were evaluated using receiver operating characteristic analysis and multivariable logistic regression. Compared with contralateral brain, meningiomas demonstrated significantly higher BF and BV, increased PS, and prolonged MTT, time-to-peak enhancement, and arterial arrival time, indicating a hypervascular and highly permeable microenvironment. Atypical meningiomas exhibited significantly higher BV, PS, and MTT than typical subtypes. BV, PS, and MTT achieved areas under the curve of 0.86, 0.81, and 0.84, respectively, for identifying atypical lesions, while a combined model incorporating BV, PS, and MTT yielded an area under the curve of 0.92 with high sensitivity and specificity. Interobserver agreement for BF, BV, PS, and MTT was excellent, with intraclass correlation coefficients ≥0.889. These findings support DCE-CT as a reproducible and informative adjunct for preoperative meningioma vascular characterization and subtype stratification.

## 1. Introduction

Intracranial meningiomas are the most common primary central nervous system tumors in adults, accounting for up to one-third of all brain neoplasms. They encompass a broad spectrum of histopathological entities and biological behaviors, ranging from indolent World Health Organization [WHO] Grade I lesions to atypical (Grade II) and anaplastic (Grade III) variants with higher recurrence rates and less favorable outcomes.^[1]^ Preoperative identification of meningioma subtype and grade is clinically relevant because it influences surgical planning, the extent of resection, anticipated intraoperative blood loss, and the need for closer postoperative surveillance or adjuvant radiotherapy. However, conventional computed tomography (CT) and magnetic resonance imaging (MRI) rely predominantly on macroscopic morphology and contrast enhancement patterns, which often do not reliably distinguish typical from atypical tumors or capture the underlying heterogeneity of tumor vascularity.^[2]^ Advances in functional and perfusion imaging have highlighted the importance of microvascular characterization in meningioma evaluation. Perfusion MRI techniques, including dynamic susceptibility contrast (DSC), arterial spin labeling (ASL), and dynamic contrast-enhanced MRI, provide quantitative information on cerebral blood flow, blood volume, and vascular permeability, parameters that correlate with neoangiogenesis and histological grade. Recent studies have demonstrated that dynamic contrast-enhanced magnetic resonance imaging (DCE-MRI)-derived time–intensity curve features and permeability metrics, in combination with diffusion parameters such as the apparent diffusion coefficient, are useful predictors for separating low-grade from high-grade meningiomas.^[3]^ ASL and DSC perfusion have also been reported to improve grading of skull base meningiomas and to aid in the differentiation of meningiomas from other extra-axial lesions.

Beyond histological grade, there is growing interest in the relationship between imaging-derived perfusion signatures and molecular subtypes of meningiomas. A recent prospective study showed that MR perfusion parameters differ across molecularly defined subgroups and are associated with proliferative indices, suggesting that vascular imaging may serve as a noninvasive surrogate for tumor biology.^[4]^ Other contemporary work has explored the use of ASL-derived cerebral blood flow to predict intraoperative blood loss in non-embolized meningiomas, underscoring the clinical relevance of quantitative vascular assessment for perioperative risk stratification.^[5]^ Collectively, these data indicate that perfusion and permeability imaging can complement structural MRI in meningioma characterization, grading, and treatment planning. In contrast to the rapidly expanding MRI literature, dynamic contrast-enhanced computed tomography (DCE-CT) and CT perfusion techniques have been less extensively investigated in meningiomas. Early work established that CT perfusion can quantify tumor blood flow, blood volume, and permeability, and that these parameters correlate with vascular density in brain tumors.^[6]^ DCE-CT offers several potential advantages, including wide availability, high temporal resolution, and straightforward integration into standard preoperative CT protocols, particularly in institutions where advanced MRI is not routinely accessible or when MRI is contraindicated. Moreover, CT-based perfusion techniques directly measure iodine contrast kinetics, providing quantitative estimates of blood flow, blood volume, mean transit time, and permeability surface area product that are conceptually analogous to DCE-MRI-derived metrics. Despite this, systematic evaluation of DCE-CT for meningioma subclassification, especially its ability to distinguish atypical from typical subtypes and to characterize subtype-specific vascular phenotypes, remains limited.^[7,8]^

There is therefore a need to clarify whether quantitative DCE-CT perfusion parameters can reliably depict the hypervascular and permeable microenvironment of meningiomas, capture relevant differences among common histopathological subtypes, and provide diagnostic information for preoperative subtype stratification. Furthermore, the clinical applicability of DCE-CT depends on the reproducibility of perfusion measurements between observers. Addressing these gaps may help establish DCE-CT as a practical, quantitative tool for preoperative meningioma subclassification and for noninvasive assessment of tumor vascularity in routine clinical settings.

## 2. Methods

### 2.1. Study design

The Third Affiliated Hospital of Soochow University. This retrospective study enrolled patients with histologically confirmed meningioma who underwent DCE-CT at our institution between October 2020 and October 2024. Eligible patients were required to meet the following inclusion criteria: a definitive diagnosis of intracranial meningioma based on postoperative pathological examination; availability of preoperative DCE-CT imaging of sufficient quality for quantitative analysis; complete clinical, radiological, and histopathological data; and no previous surgical, radiotherapeutic, or pharmacologic treatment that could alter tumor perfusion characteristics. Patients were excluded if they: had recurrent meningioma or a history of prior intracranial tumor surgery; exhibited poor-quality or incomplete imaging that precluded quantitative perfusion assessment; had coexisting intracranial lesions that interfered with imaging interpretation; or had severe cardiac, renal, or allergic conditions contraindicating iodinated contrast administration. The study adhered to the ethical principles of the Declaration of Helsinki and received approval from the institutional medical ethics committee. Written informed consent was obtained from all surviving participants or from their legal guardians when applicable. Four patients in the cohort were diagnosed with WHO Grade III (anaplastic) meningiomas. Because of the very limited number of Grade III cases, separate statistical analysis of this subgroup was not feasible. To avoid introducing statistical bias or unstable estimates, Grade III tumors were not included in the main comparative analysis between typical (Grade I) and atypical (Grade II) meningiomas.

### 2.2. Imaging protocol

DCE-CT examinations were performed using a multi-detector CT scanner equipped with perfusion imaging capability. All patients were instructed to avoid movement and remained in the supine position with the head immobilized during acquisition. A non-contrast head CT was initially obtained to identify the lesion location and to guide the subsequent perfusion scan. DCE-CT imaging was then performed using the following standardized parameters: tube voltage 80 to 100 kVp, tube current 100 to 200 mAs, collimation 64 × 0.6 mm, rotation time 0.5 to 1.0 seconds, slice thickness 5 mm, and a temporal sampling interval of 1 to 2 seconds. The perfusion scan covered the entire tumor level or the region demonstrating maximal enhancement on the baseline images. A bolus of nonionic iodinated contrast medium (iodine concentration 300–370 mg/mL) was injected through an antecubital vein using a power injector at a rate of 4 to 5 mL/s, followed by a 20 to 30 mL saline flush. The total contrast volume was 50 to 70 mL, adjusted according to patient body weight. Image acquisition commenced 3 to 5 seconds after the initiation of contrast injection and continued for 45 to 60 seconds to capture the arterial, capillary, and venous phases of tumor enhancement. All raw perfusion data were transferred to a dedicated workstation for post-processing. Motion correction was applied when necessary, and arterial input function was selected from the ipsilateral middle cerebral artery. Quantitative perfusion maps, including blood flow (BF), blood volume (BV), mean transit time (MTT), and permeability surface area product (PS), were generated using a deconvolution-based algorithm. Regions of interest (ROIs) were manually placed by 2 experienced neuroradiologists, avoiding areas of calcification, necrosis, hemorrhage, and adjacent normal tissue.

### 2.3. Data collection

Demographic, clinical, imaging, and pathological data were retrospectively collected from the institutional electronic medical record and picture archiving and communication system for all eligible patients. Demographic variables included age and sex. Tumor-related clinical information included the date of diagnosis and preoperative imaging work-up. For each patient, preoperative non-contrast CT and DCE-CT images were reviewed to determine tumor location and size. Tumor location was categorized as convexity, skull base, parasagittal region, falx, or posterior fossa according to the predominant site of attachment. The maximum tumor diameter was measured on axial or coronal contrast-enhanced images and classified into 3 subgroups (<3.0 cm, 3.0–5.0 cm, and >5.0 cm).

Morphological CT features were systematically recorded, including the presence or absence of intratumoral calcification, cystic or necrotic components, peritumoral edema, and the dural tail sign. Peritumoral edema was assessed on preoperative CT and graded as present or absent based on the visualization of low-attenuation changes in the adjacent brain parenchyma. The dural tail sign was defined as linear or tapered enhancement of the dura mater extending from the tumor margin on contrast-enhanced images.

Histopathological data were obtained from surgical pathology reports. All tumors were classified according to the WHO classification of central nervous system tumors into Grade I, Grade II, or Grade III meningiomas. Histological subtypes were further recorded and grouped into the 5 most common variants observed in this cohort: meningothelial, transitional, fibrous, psammomatous, and atypical meningiomas. For subsequent diagnostic performance analyses, histological subtypes were dichotomized into typical meningiomas (WHO Grade I; meningothelial, transitional, fibrous, and psammomatous) and atypical meningiomas (WHO Grade II).

Quantitative perfusion parameters were derived from preoperative DCE-CT examinations using a dedicated perfusion analysis workstation. For each tumor, ROIs were manually placed within the solid, visibly enhancing portion of the lesion, avoiding areas of calcification, cystic or necrotic degeneration, hemorrhage, and adjacent normal brain tissue. A corresponding ROI was placed in contralateral normal-appearing brain parenchyma at the same or nearest axial level to serve as internal reference tissue. The following perfusion metrics were extracted from the time–attenuation curves for both tumor and contralateral ROIs: BF (mL/min/100 g), BV (mL/100 g), MTT (s), PS (mL/min/100 g), time-to-peak enhancement (TTP, s), and arterial arrival time (AAT, s). All measurements were independently performed by 2 experienced radiologists who were blinded to the histopathological classification and clinical outcomes. For the main analyses, the average value of the 2 readers was used, while interobserver agreement for BF, BV, PS, and MTT was evaluated using intraclass correlation coefficients.

### 2.4. Statistical analysis

All statistical analyses were performed using IBM SPSS Statistics for Windows, version 28.0 (IBM Corp., Armonk). A two-sided *P* value <.05 was considered statistically significant. Continuous variables were expressed as mean ± standard deviation and categorical variables as counts and percentages. Perfusion parameters between meningioma tissue and contralateral normal brain were compared using paired *t* tests. Differences in BF, BV, PS, MTT, and TTP among histopathological subtypes were assessed with one-way analysis of variance. Receiver operating characteristic (ROC) curves were constructed to evaluate the diagnostic performance of BF, BV, PS, MTT, and TTP for differentiating atypical from typical meningiomas; the area under the curve (AUC), 95% confidence intervals, and optimal cutoff values based on the Youden index were calculated, and the corresponding sensitivity, specificity, positive predictive value (PPV), and negative predictive value (NPV) were derived. A multivariable logistic regression model including BV, PS, and MTT was used to generate a combined predictive probability and its AUC and diagnostic indices. Interobserver agreement for BF, BV, PS, and MTT was quantified using intraclass correlation coefficients (ICC) with 95% confidence intervals.

## 3. Results

### 3.1. Baseline characteristics

A total of 68 patients diagnosed with intracranial meningioma were included in this study. The mean age of the cohort was 52.3 ± 11.8 years, with a range of 29 to 76 years, and women accounted for 64.7% of the study population. Tumors were most frequently located at the cerebral convexity (32.4%) and skull base (26.5%), followed by the parasagittal region (17.6%), posterior fossa (13.2%), and falx (10.3%). The maximum tumor diameter measured 3.0 to 5.0 cm in 48.5% of patients, whereas 27.9% had tumors smaller than 3.0 cm and 23.5% had lesions larger than 5.0 cm. Histopathological assessment demonstrated that the majority of cases were WHO Grade I meningiomas (76.5%), with Grade II and Grade III tumors representing 17.6% and 5.9% of the cohort, respectively. Among the histological subtypes, meningothelial (29.4%) and transitional (26.5%) variants were most prevalent, followed by fibrous (22.1%), psammomatous (8.8%), and atypical forms (13.2%). Calcification was identified in 25.0% of tumors, while cystic or necrotic components were present in 13.2% of cases. Peritumoral edema was observed in 60.3% of patients. The dural tail sign was documented in 79.4% of the cohort (Table 1).

**Table 1:** Baseline characteristics of the study cohort.

Variable	Category/summary	n (%) or mean ± SD
Age (yr)	Mean ± SD (range)	52.3 ± 11.8 (29–76)
Sex	Female	44 (64.7)
	Male	24 (35.3)
Tumor location	Convexity	22 (32.4)
	Skull base	18 (26.5)
	Parasagittal	12 (17.6)
	Falx	7 (10.3)
	Posterior fossa	9 (13.2)
Maximum tumor diameter	<3.0 cm	19 (27.9)
	3.0–5.0 cm	33 (48.5)
	>5.0 cm	16 (23.5)
WHO grade	Grade I	52 (76.5)
	Grade II	12 (17.6)
	Grade III	4 (5.9)
Histopathological subtype	Meningothelial	20 (29.4)
	Transitional	18 (26.5)
	Fibrous	15 (22.1)
	Psammomatous	6 (8.8)
	Atypical	9 (13.2)
Calcification	Present	17 (25.0)
	Absent	51 (75.0)
Cystic/necrotic component	Present	9 (13.2)
	Absent	59 (86.8)
Peritumoral edema	Present	41 (60.3)
	Absent	27 (39.7)
Dural tail sign	Present	54 (79.4)
	Absent	14 (20.6)

SD = standard deviation, WHO = World Health Organization.

### 3.2. DCE-CT quantitative perfusion parameters

Quantitative DCE-CT analysis showed significant perfusion differences between meningioma tissue and contralateral normal-appearing brain parenchyma. Tumor blood flow was markedly higher than that of healthy brain (59.83 ± 10.61 vs 30.83 ± 5.26 mL/min/100 g; mean difference 29.00, 95% confidence interval [CI] 26.55–31.45; *t* = 23.58; *P* < .001), and tumor blood volume demonstrated a similar increase (4.17 ± 0.72 vs 2.19 ± 0.37 mL/100 g; mean difference 1.98, 95% CI 1.83–2.14; *t* = 26.26; *P* < .001). Parameters reflecting contrast transit were also altered in tumor tissue. Mean transit time was prolonged in meningiomas compared with contralateral brain (6.21 ± 1.33 vs 4.28 ± 0.84 seconds; mean difference 1.93, 95% CI 1.68–2.18; *t* = 15.60; *P* < .001). The permeability surface area product, indicating microvascular permeability, was substantially elevated in tumors (1.76 ± 0.44 vs 0.59 ± 0.13 mL/min/100 g; mean difference 1.17, 95% CI 1.08–1.27; *t* = 23.79; *P* < .001). In addition, enhancement timing parameters were delayed in tumor regions. Time-to-peak enhancement was longer in meningioma tissue than in healthy brain (21.96 ± 4.61 vs 17.63 ± 3.09 seconds; mean difference 4.33, 95% CI 3.28–5.38; *t* = 8.23; *P* < .001), and AAT was similarly increased (3.26 ± 0.64 vs 2.36 ± 0.48 seconds; mean difference 0.90, 95% CI 0.76–1.04; *t* = 12.88; *P* < .001). These findings indicate that meningiomas exhibit higher blood flow and blood volume, increased vascular permeability, and delayed contrast transit compared with contralateral normal brain, supporting the utility of DCE-CT perfusion parameters for characterizing tumor vascularity (Table 2).

**Table 2:** Quantitative DCE-CT perfusion parameters of meningiomas and contralateral healthy brain tissue.

Parameter	Tumor (mean ± SD)	Contralateral healthy brain (mean ± SD)	Mean difference (tumor − healthy), 95% CI	*t* value	*P* value
BF (mL/min/100 g)	59.83 ± 10.61	30.83 ± 5.26	29.00 (26.55–31.45)	23.58	<.001
BV (mL/100 g)	4.17 ± 0.72	2.19 ± 0.37	1.98 (1.83–2.14)	26.26	<.001
MTT (s)	6.21 ± 1.33	4.28 ± 0.84	1.93 (1.68–2.18)	15.60	<.001
PS (mL/min/100 g)	1.76 ± 0.44	0.59 ± 0.13	1.17 (1.08–1.27)	23.79	<.001
TTP (s)	21.96 ± 4.61	17.63 ± 3.09	4.33 (3.28–5.38)	8.23	<.001
AAT (s)	3.26 ± 0.64	2.36 ± 0.48	0.90 (0.76–1.04)	12.88	<.001

AAT = arterial arrival time, BF = blood flow, BV = blood volume, CI = confidence interval, DCE-CT = dynamic contrast-enhanced computed tomography, MTT = mean transit time, PS = permeability surface area product, SD = standard deviation, TTP = time-to-peak enhancement.

### 3.3. Comparison of perfusion parameters among meningioma subtypes

DCE-CT perfusion analysis demonstrated subtype-related differences in vascular and hemodynamic parameters among the 5 most common histopathological meningioma variants. Atypical meningiomas showed the highest BF, BV, PS, and MTT values, whereas psammomatous tumors tended to have lower BV, PS, and MTT. Although BF was numerically greater in atypical lesions (66.63 ± 8.85 mL/min/100 g) than in meningothelial, transitional, fibrous, and psammomatous subtypes (55.80–59.73 mL/min/100 g), the overall difference in BF did not reach statistical significance (*F* = 1.94, *P* = .118). In contrast, BV varied significantly across subtypes (*F* = 7.43, *P* < .001). Atypical meningiomas exhibited the highest BV (4.97 ± 0.57 mL/100 g), whereas psammomatous tumors showed the lowest BV (3.48 ± 0.75 mL/100 g), with meningothelial, transitional, and fibrous subtypes presenting intermediate values. PS also differed significantly between subtypes (*F* = 3.31, *P* = .017), with atypical lesions demonstrating increased permeability (2.05 ± 0.58 mL/min/100 g) compared with typical Grade I subtypes (1.43–1.65 mL/min/100 g). MTT showed a significant prolongation in atypical meningiomas (7.15 ± 0.88 seconds) compared with the other subtypes, which ranged from 5.18 to 6.21 seconds, resulting in a significant overall difference (*F* = 6.67, *P* < .001). TTP exhibited numerical variation, with slightly longer times in fibrous, psammomatous, and atypical tumors; however, the overall difference in TTP among subtypes was not statistically significant (*F* = 1.33, *P* = .273) (Table 3).

**Table 3:** Comparison of DCE-CT perfusion parameters among meningioma subtypes.

Subtype/parameter	BF (mL/min/100 g) mean ± SD	BV (mL/100 g) mean ± SD	PS (mL/min/100 g) Mean ± SD	MTT (s) mean ± SD	TTP (s) mean ± SD
Meningothelial (n = 20)	57.12 ± 8.01	4.09 ± 0.51	1.62 ± 0.28	5.74 ± 0.83	19.74 ± 4.13
Transitional (n = 14)	59.24 ± 11.94	4.21 ± 0.62	1.65 ± 0.37	6.21 ± 0.91	21.79 ± 2.53
Fibrous (n = 8)	55.80 ± 7.25	3.89 ± 0.44	1.60 ± 0.27	5.81 ± 0.69	22.51 ± 7.46
Psammomatous (n = 6)	59.73 ± 9.79	3.48 ± 0.75	1.43 ± 0.25	5.18 ± 0.65	22.68 ± 3.66
Atypical (n = 9)	66.63 ± 8.85	4.97 ± 0.57	2.05 ± 0.58	7.15 ± 0.88	23.12 ± 4.28
*F* value	1.94	7.43	3.31	6.67	1.33
*P* value	.118	<.001	.017	<.001	.273

BF = blood flow, BV = blood volume, DCE-CT = dynamic contrast-enhanced computed tomography, MTT = mean transit time, PS = permeability surface area product, SD = standard deviation, TTP = time-to-peak enhancement.

### 3.4. Diagnostic performance for meningioma subtype differentiation

For diagnostic performance analysis, histopathological subtypes were dichotomized into typical meningiomas (WHO Grade I; meningothelial, transitional, fibrous, and psammomatous) and atypical meningiomas (WHO Grade II). Receiver operating characteristic curve analysis showed that DCE-CT perfusion parameters provided varying discriminatory ability for identifying atypical lesions. Among the single parameters, BV, PS, and MTT demonstrated the highest diagnostic accuracy. BV yielded an AUC of 0.86 (95% CI, 0.76–0.95; *P* < .001), with an optimal cutoff value of >4.50 mL/100 g based on the Youden index, resulting in a sensitivity of 77.8% and a specificity of 83.3%, and corresponding PPV and NPV values of 50.0% and 94.4%, respectively. PS also showed good discriminatory performance (AUC 0.81, 95% CI, 0.69–0.93; *P* < .001) at an optimal threshold of >1.85 mL/min/100 g, with a sensitivity of 77.8%, specificity of 79.2%, PPV of 46.7%, and NPV of 93.0%. MTT exhibited an AUC of 0.84 (95% CI, 0.74–0.94; *P* < .001), and a cutoff value of >6.70 seconds provided high sensitivity (88.9%) with acceptable specificity (72.9%), yielding a PPV of 44.4% and an NPV of 96.7%. BF showed moderate diagnostic value (AUC 0.73, 95% CI, 0.60–0.86; *P* = .004), whereas TTP achieved only limited discriminatory capacity (AUC 0.63, 95% CI, 0.49–0.78; *P* = .090), indicating that TTP alone was not sufficient for reliable subtype differentiation. A multivariable logistic regression model incorporating BV, PS, and MTT achieved the highest diagnostic accuracy, with an AUC of 0.92 (95% CI, 0.85–0.99; *P* < .001). Using an optimal probability threshold of 0.35, the combined model yielded a sensitivity of 88.9% and a specificity of 87.5%, with a PPV of 61.5% and an NPV of 97.9% (Table 4).

**Table 4:** Diagnostic performance of DCE-CT perfusion parameters for differentiating atypical from typical meningiomas.

Parameter/ Model	AUC (95% CI)	Optimal cutoff	Sensitivity (%)	Specificity (%)	PPV (%)	NPV (%)
BF (mL/min/100 g)	0.73 (0.60–0.86)	> 62.0	66.7	72.9	36.4	90.9
BV (mL/100 g)	0.86 (0.76–0.95)	> 4.50	77.8	83.3	50.0	94.4
PS (mL/min/100 g)	0.81 (0.69–0.93)	> 1.85	77.8	79.2	46.7	93.0
MTT (s)	0.84 (0.74–0.94)	> 6.70	88.9	72.9	44.4	96.7
TTP (s)	0.63 (0.49–0.78)	> 21.5	66.7	62.5	29.6	89.7
Combined model (BV + PS + MTT)	0.92 (0.85–0.99)	Predicted probability > 0.35	88.9	87.5	61.5	97.9

AUC = area under the receiver operating characteristic curve, BF = blood flow, BV = blood volume, CI = confidence interval, DCE-CT = dynamic contrast-enhanced computed tomography, MTT = mean transit time, NPV = negative predictive value, PPV = positive predictive value, PS = permeability surface area product, TTP = time-to-peak enhancement.

### 3.5. Interobserver agreement

Interobserver reproducibility of DCE-CT perfusion measurements between the 2 radiologists was high for all evaluated parameters. The ICC for BF was 0.936 (95% CI, 0.902–0.960; *P* < .001), indicating very good agreement between observers. BV showed a similarly high level of consistency, with an ICC of 0.921 (95% CI, 0.882–0.949; *P* < .001). PS also demonstrated robust interobserver reliability (ICC 0.908; 95% CI, 0.864–0.940; *P* < .001). MTT yielded an ICC of 0.889 (95% CI, 0.837–0.927; *P* < .001), confirming stable measurement agreement (Table 5).

**Table 5:** Interobserver agreement for DCE-CT perfusion measurements between 2 radiologists.

Parameter	ICC (95% CI)	*P* value
BF (mL/min/100 g)	0.936 (0.902–0.960)	<.001
BV (mL/100 g)	0.921 (0.882–0.949)	<.001
PS (mL/min/100 g)	0.908 (0.864–0.940)	<.001
MTT (s)	0.889 (0.837–0.927)	<.001

BF = blood flow, BV = blood volume, CI = confidence interval, DCE-CT = dynamic contrast-enhanced computed tomography, ICC = intraclass correlation coefficient, MTT = mean transit time, PS = permeability surface area product.

## 4. Discussion

This study evaluated the ability of dynamic contrast-enhanced computed tomography to characterize vascular properties of intracranial meningiomas and to distinguish atypical from typical subtypes using quantitative perfusion metrics. Several main findings emerge. First, compared with contralateral normal-appearing brain parenchyma, meningiomas demonstrated higher blood flow and blood volume, increased permeability, and prolonged contrast transit, confirming a distinct hypervascular and leaky microcirculation profile. Second, among histopathological subtypes, atypical meningiomas were characterized by higher BV, PS, and MTT than typical Grade I variants, indicating more pronounced microvascular proliferation and barrier disruption. Third, BV, PS, and MTT showed good diagnostic performance for identifying atypical lesions, and a combined model integrating these parameters achieved excellent discrimination with an AUC of 0.92. Finally, interobserver agreement for DCE-CT-derived perfusion metrics was high, supporting the reproducibility of the measurement protocol.

The substantial elevation of BF and BV in meningiomas relative to contralateral brain is consistent with the known rich vascular supply of these extra-axial tumors. Meningiomas are predominantly supplied by branches of the external carotid and meningeal arteries, which lack a normal blood–brain barrier. This vascular anatomy is expected to yield higher microvascular density and increased intravascular volume, reflected by elevated BV, and to facilitate rapid contrast inflow, reflected in increased BF. The concomitant prolongation of MTT and elevation of PS suggest that, in addition to increased vessel number, there is altered microvascular architecture with tortuous channels and impaired venous drainage, together with structurally abnormal endothelium and basement membrane that permit substantial leakage of contrast into the interstitium.^[9,10]^ The delayed TTP and prolonged AAT in tumor tissue support a more complex and inefficient transit pattern, in which contrast arrival and peak enhancement are slowed despite high flow and volume, likely due to heterogeneous vascular pathways and variable resistance.

Subtype analysis further refines this interpretation. Atypical meningiomas in this cohort presented with the highest BV and PS and significantly longer MTT compared with Grade I variants. These findings are compatible with more advanced neovascularization and structural disruption of the vascular wall in higher-grade lesions. Increased BV may represent both angiogenesis and vasodilation, while elevated PS reflects a more permeable microvasculature, possibly related to microscopic necrosis, endothelial fenestration, and degradation of extracellular matrix. Prolonged MTT suggests sluggish microcirculation and inefficient washout, which may be associated with increased interstitial pressure and altered venous outflow. In contrast, psammomatous meningiomas, which often contain abundant calcified psammoma bodies and relatively less proliferative tissue, demonstrated lower BV, PS, and MTT, consistent with a more mature and less hyperplastic vascular bed.

The absence of a statistically significant overall difference in BF among subtypes, despite numerically higher BF in atypical lesions, indicates that flow alone may be less sensitive to grade-related changes than volume and permeability measures. BF is influenced by arterial inflow and systemic hemodynamics, whereas BV and PS more directly reflect tumor microvascular remodeling and barrier integrity. TTP did not differ significantly among subtypes, which may reflect the combined and sometimes opposing effects of high flow, high volume, and delayed washout on the time–intensity curve. These observations support the view that a multiparametric assessment incorporating BV, PS, and MTT may more accurately capture biologically relevant differences in meningioma vascularity than any single timing parameter.

The ROC analysis demonstrates that DCE-CT-derived BV, PS, and MTT each provide meaningful discrimination between atypical and typical meningiomas. BV and MTT, in particular, yielded AUCs above 0.80, with cutoff values (>4.50 mL/100 g for BV and > 6.70 seconds for MTT) that balanced sensitivity and specificity at clinically acceptable levels. High NPVs for these parameters (above 94%) suggest that lesions below these thresholds are unlikely to be atypical, which may be useful when the clinical question is whether more aggressive biological behavior is probable. PS also offered good discrimination, again highlighting the central role of permeability in meningioma grading. The multivariable logistic regression model combining BV, PS, and MTT achieved an AUC of 0.92, with both sensitivity and specificity close to 90%. This result suggests that the parameters encode partly complementary information on vascular quantity, leakiness, and transit kinetics. The relatively high NPV of the combined model (97.9%) indicates that a low predicted probability of atypical histology could help reassure clinicians when planning surgical strategy or follow-up, although the moderate PPV underscores that histopathology remains necessary for definitive grading. The excellent intraclass correlation coefficients observed for BF, BV, PS, and MTT confirm that, under a standardized acquisition and analysis protocol, DCE-CT perfusion metrics are reproducible between readers and are suitable for clinical and research use.

Recent work using advanced MRI has also highlighted the value of perfusion and permeability metrics for meningioma characterization. Panigrahi et al reported that advanced MRI, including perfusion imaging and diffusion metrics, could differentiate various meningioma subtypes and distinguish typical from atypical lesions; higher perfusion values were generally associated with more aggressive variants.^[11]^ Utomo et al demonstrated that DCE-MRI parameters in combination with apparent diffusion coefficient values were significant predictors for separating low-grade from high-grade meningiomas, with time–intensity curve-derived metrics and maximal contrast enhancement ratio showing good diagnostic performance.^[3]^ These MRI-based results are consistent with the present DCE-CT findings, in which parameters reflecting blood volume and permeability discriminated atypical from typical tumors with reasonably high AUCs. Ota et al examined diffusion-weighted imaging and DCE-MRI in brain tumors and found that perfusion-derived pharmacokinetic parameters correlated with tumor grade and proliferative indices, suggesting that microvascular permeability metrics are proxies for underlying tumor biology.^[12]^ More recently, Eissa et al evaluated combined arterial spin labeling and DSC perfusion to grade skull base meningiomas, reporting that relative cerebral blood volume and flow values were significantly higher in high-grade lesions and that perfusion imaging improved noninvasive grading.^[13]^ Jafarpour et al further extended perfusion analysis to molecular subtypes, demonstrating significant associations between perfusion parameters, WHO grade, and molecular features, underscoring the link between meningioma vascular phenotype and underlying biology.^[4]^

These studies collectively support the concept that perfusion and permeability imaging, most commonly performed with MRI, can aid in preoperative grading and subtyping of meningiomas. The present results extend this concept to DCE-CT, showing that CT-based quantitative perfusion metrics can similarly differentiate atypical from typical meningiomas and characterize vascular heterogeneity across histological subtypes. While DCE-CT has lower soft-tissue contrast than MRI and includes ionizing radiation, it may be more available in some settings and can be integrated into routine preoperative CT workflows. The observed diagnostic performance of BV, PS, and MTT is comparable to that reported for MRI-based perfusion parameters in recent series, suggesting that, when MRI is contraindicated or unavailable, DCE-CT offers a viable alternative for vascular characterization and subtype risk stratification.^[14]^ Furthermore, recent reviews have emphasized the role of advanced neuroimaging and quantitative perfusion in integrating structural, functional, and molecular information for meningioma management.^[15]^ Within this broader context, the present study supports the incorporation of perfusion-based biomarkers into multiparametric imaging strategies and suggests that CT-based metrics can complement MRI-based approaches in comprehensive preoperative assessment. An important consideration for DCE-CT is radiation exposure. Although modern CT perfusion protocols employ dose-reduction strategies such as lower tube voltage, optimized temporal sampling, and limited scan coverage, the examination still involves ionizing radiation and iodinated contrast administration. Therefore, the clinical use of DCE-CT should be carefully justified. In practice, this technique may be particularly valuable in specific clinical scenarios, including patients with contraindications to MRI (such as implanted ferromagnetic devices or severe claustrophobia), emergency situations in which rapid imaging is required, and institutions where advanced MRI perfusion techniques are not readily available. In these contexts, DCE-CT may provide a practical and accessible alternative for evaluating tumor vascularity and estimating the likelihood of atypical meningioma prior to surgery.

From a clinical perspective, the present findings suggest that DCE-CT perfusion imaging can provide useful adjunctive information beyond conventional morphology for meningioma evaluation. Quantitative BV, PS, and MTT values may help identify lesions at higher risk of atypical histology before surgery, supporting decisions regarding surgical planning, the extent of resection, the need for closer postoperative surveillance, and discussion of potential adjuvant therapy. High NPVs for single parameters and for the combined model indicate that low perfusion-based risk estimates may help to stratify patients toward standard follow-up, whereas markedly elevated values could prompt more intensive management strategies. In institutions where MRI perfusion is not routinely available, DCE-CT may represent a practical and reproducible method to incorporate vascular characterization into routine care. This study has several strengths. All patients underwent standardized preoperative DCE-CT with quantitative perfusion analysis, and perfusion metrics were compared not only with contralateral brain tissue but also across histopathological subtypes and grades. Diagnostic performance was evaluated using ROC analysis with explicit calculation of cutoff values, sensitivity, specificity, PPV, and NPV, and a multivariable model was developed to explore the added value of combining perfusion parameters. Interobserver reproducibility was formally assessed using ICCs, demonstrating robust agreement between radiologists and supporting the reliability of the measurement protocol.

Several limitations should be considered when interpreting the findings. The retrospective single-center design is subject to selection bias, and the relatively modest sample size, particularly for atypical meningiomas and less common subtypes, may limit the stability of cutoff values and the generalizability of the diagnostic model. The dichotomization into typical and atypical groups did not include a sufficient number of WHO Grade III tumors to permit a separate analysis of highly aggressive disease. DCE-CT involves ionizing radiation and iodinated contrast, which may restrict its application in certain patient populations compared with MRI-based perfusion techniques. In addition, only intratumoral ROIs were evaluated; perfusion characteristics of peritumoral edema and feeding arteries, which may also carry prognostic information, were not analyzed. External validation of the combined logistic model in independent cohorts and comparison with MRI perfusion metrics were beyond the scope of the current work but would be necessary before wider clinical adoption. A small number of WHO Grade III meningiomas were present in the cohort. Because this subgroup included only 4 cases, separate statistical analysis was not feasible and these cases were excluded from the main diagnostic analysis. Consequently, the findings of this study mainly apply to the differentiation between typical (Grade I) and atypical (Grade II) meningiomas. The exclusion of WHO Grade III meningiomas from the main analysis may also limit the generalizability of the findings. The diagnostic model developed in this study was specifically designed to differentiate typical (Grade I) from atypical (Grade II) meningiomas, and its performance in identifying highly aggressive Grade III tumors remains unclear. Future investigations including a larger number of anaplastic meningiomas are required to determine whether DCE-CT perfusion parameters can reliably characterize the full spectrum of meningioma grades.

The relatively small number of atypical meningiomas in the present cohort should be acknowledged. Higher-grade meningiomas represent a minority of cases in routine clinical practice, which limited the available sample size in this retrospective study. Although statistically significant differences in several perfusion parameters were observed, the limited number of Grade II tumors may affect the robustness of cutoff values and predictive modeling. Future multicenter studies with larger and more balanced cohorts are needed to further validate the diagnostic performance of DCE-CT perfusion parameters.

## 5. Conclusion

DCE-CT–derived perfusion parameters quantitatively demonstrated that meningiomas exhibit increased blood flow, blood volume, permeability, and delayed contrast transit compared with normal brain, reflecting their characteristic hypervascularity. Subtype analysis showed that BV, PS, and MTT effectively differentiate atypical from typical meningiomas, and a combined perfusion model achieved high diagnostic accuracy with excellent reproducibility, supporting DCE-CT as a useful adjunct for preoperative meningioma subclassification.

## Author contributions

**Conceptualization:** Shanmei Liu, Tianchun Wang, Wei Li, Haitao Lu.

**Data curation:** Shanmei Liu, Tianchun Wang, Wei Li, Haitao Lu.

**Formal analysis:** Shanmei Liu, Tianchun Wang, Wei Li, Haitao Lu.

**Funding acquisition:** Shanmei Liu, Haitao Lu.

**Investigation:** Haitao Lu.

**Writing – original draft:** Shanmei Liu, Haitao Lu.

**Writing – review & editing:** Shanmei Liu, Haitao Lu.
